# Assessment and prevention of hypoglycaemia in primary care among U.S. Veterans: a mixed methods study

**DOI:** 10.1016/j.lana.2023.100641

**Published:** 2023-11-24

**Authors:** Scott J. Pilla, Kayla A. Meza, Mary Catherine Beach, Judith A. Long, Howard S. Gordon, Jeffrey T. Bates, Donna L. Washington, Barbara G. Bokhour, Anais Tuepker, Somnath Saha, Nisa M. Maruthur

**Affiliations:** a Department of Medicine, Division of General Internal Medicine, Johns Hopkins University School of Medicine, Baltimore, MD, USA; b Department of Health Policy and Management, Johns Hopkins University Bloomberg School of Public Health, Baltimore, MD, USA; c Welch Center for Prevention, Epidemiology and Clinical Research, Johns Hopkins University, Baltimore, MD, USA; d University of Colorado Anschutz Medical Campus, Aurora, CO, USA; e Department of Health, Behavior & Society, Johns Hopkins University Bloomberg School of Public Health, Baltimore, MD, USA; f Corporal Michael J. Cresenz VA Medical Center, Philadelphia, PA, USA; g Division of General Internal Medicine, University of Pennsylvania, Perelman School of Medicine, Philadelphia, PA, USA; h Jesse Brown VA Medical Center, Chicago, IL, USA; i Division of Academic Internal Medicine and Geriatrics, Department of Medicine, University of Illinois Chicago College of Medicine, Chicago, IL, USA; j Michael E. DeBakey VA Medical Center, Houston, TX, USA; k Baylor College of Medicine, Houston, TX, USA; l VA Health Services Research and Development Center for the Study of Healthcare Innovation, Implementation, and Policy, VA Greater Los Angeles Healthcare System, Los Angeles, CA, USA; m Division of General Internal Medicine and Health Services Research, Department of Medicine, Geffen School of Medicine, University of California, Los Angeles, Los Angeles, CA, USA; n Center for Healthcare Organization and Implementation Research, VA Bedford Health Care System, Bedford, MA, USA; o Department of Population and Quantitative Health Sciences, University of Massachusetts Chan Medical School, Worcester, MA, USA; p Center to Improve Veteran Involvement in Care, VA Portland Health Care System, Portland, OR, USA; q Department of Family Medicine, Oregon Health & Science University, Portland, OR, USA; r Department of Epidemiology, Johns Hopkins University Bloomberg School of Public Health, Baltimore, MD, USA

**Keywords:** Diabetes mellitus, Hypoglycemia, Drug-related side effects and adverse reactions, Deprescriptions, Health education

## Abstract

**Background:**

Hypoglycaemia from diabetes treatment causes morbidity and lower quality of life, and prevention should be routinely addressed in clinical visits.

**Methods:**

This mixed methods study evaluated how primary care providers (PCPs) assess for and prevent hypoglycaemia by analyzing audio-recorded visits from five Veterans Affairs medical centres in the US. Two investigators independently coded visit dialogue to classify discussions of hypoglycaemia history, anticipatory guidance, and adjustments to hypoglycaemia-causing medications according to diabetes guidelines.

**Findings:**

There were 242 patients (one PCP visit per patient) and 49 PCPs. Two thirds of patients were treated with insulin and 40% with sulfonylureas. Hypoglycaemia history was discussed in 78/242 visits (32%). PCPs provided hypoglycaemia anticipatory guidance in 50 visits (21%) that focused on holding diabetes medications while fasting and carrying glucose tabs; avoiding driving and glucagon were not discussed. Hypoglycaemia-causing medications were de-intensified or adjusted more often (p < 0.001) when the patient reported a history of hypoglycaemia (15/51 visits, 29%) than when the patient reported no hypoglycaemia or it was not discussed (6/191 visits, 3%). Haemoglobin A1c (HbA1c) was not associated with diabetes medication adjustment, and only 5/12 patients (42%) who reported hypoglycaemia with HbA1c <7.0% had medications de-intensified or adjusted.

**Interpretation:**

PCPs discussed hypoglycaemia in one-third of visits for at-risk patients and provided limited hypoglycaemia anticipatory guidance. De-intensifying or adjusting hypoglycaemia-causing medications did not occur routinely after reported hypoglycaemia with HbA1c <7.0%. Routine hypoglycaemia assessment and provision of diabetes self-management education are needed to achieve guideline-concordant hypoglycaemia prevention.

**Funding:**

10.13039/100000738U.S. Department of Veterans Affairs and 10.13039/100000062National Institute of Diabetes and Digestive and Kidney Diseases (NIDDK).

**Research in context**

**Evidence before this study**

Effective diabetes care for patients at risk for hypoglycaemia includes routine assessment of hypoglycaemia history and evidence-based prevention practices including patient education and medication adjustment. Despite this, there are limited studies examining how primary care providers perform these actions in practice. We conducted a PubMed search with the Mesh terms for “Hypoglycaemia”, “Primary Health Care”, AND “Diabetes Mellitus” from inception through July 3, 2023. We found only two relevant studies that were both single-centre cohorts and had limited data on providers’ hypoglycaemia prevention practices.

**Added value of this study**

This is the first large, multi-centre cohort examining hypoglycaemia prevention in primary care, and uniquely combines qualitative analyses of visit dialogue with data from healthcare records to gain a comprehensive understanding of hypoglycaemia prevention practices.

**Implications of all the available evidence**

Primary care providers do not routinely assess hypoglycaemia history for patients at risk for hypoglycaemia, and provide limited anticipatory guidance, especially around treatment. Primary care providers do not routinely de-intensify or adjust hypoglycaemia-causing medications for patients with a history of hypoglycaemia and tight glycemic control, as is recommended in diabetes guidelines. Therefore, additional resources are needed to achieve hypoglycaemia assessment and prevention in primary care.

## Introduction

Diabetes treatment with sulfonylureas, meglitinides, and especially insulin, poses a substantial risk for hypoglycaemia.^1^ Hypoglycaemia is defined as blood glucose less than 70 mg/dl regardless of symptoms.^2^ Level 2 hypoglycaemia (glucose <54 mg/dl) requires immediate action to correct glucose levels, and level 3 hypoglycaemia (altered mental and/or physical status requiring assistance) is a medical emergency. Hypoglycemic events (incidents of hypoglycaemia) are very common among people with diabetes, although most epidemiologic studies capture only level 3 events resulting in emergency department utilization or hospitalization.^3^, ^4^, ^5^ Nevertheless, hypoglycemic events cause more hospitalizations than hyperglycemia and are associated with falls, motor vehicle accidents, cardiovascular morbidity, and a lower quality of life.^3^, ^4^, ^5^

For patients taking hypoglycaemia-causing medications, diabetes guidelines recommend assessing hypoglycaemia history and risk factors at each clinical visit.^2^ Hypoglycaemia risk assessment is crucial for selecting glycemic targets and modifying treatment to prevent recurrent hypoglycemic events.^2^ Guidelines also recommend that patients at risk for hypoglycaemia receive routine counselling on hypoglycaemia prevention and treatment.^2^ As most diabetes care in the U.S. occurs in the primary care setting, primary care providers (PCPs) have a central role in hypoglycaemia prevention.^6^

Previously, we performed a study examining hypoglycaemia communication between PCPs and at-risk patients at a single institution, which found that hypoglycaemia history was discussed in 24% of visits.^7^ In this study, we aimed to evaluate how PCPs assess for hypoglycaemia and take appropriate preventive action for at-risk patients in a large, multicenter cohort.

## Methods

### Study design, setting and participants

In this mixed methods study, we performed qualitative and quantitative analyses of data collected in the Opening the Black Box of Cultural Competence (Black Box) study. Black Box was an observational study evaluating patient-provider communication among patients with diabetes. Patients and their PCPs were recruited from five Veterans Affairs medical centres (VAMCs) in four geographically diverse locations: Chicago (Jesse Brown VAMC), Houston (Michael E. DeBakey VAMC), Philadelphia (Corporal Michael J. Crescenz VAMC), and Los Angeles (West Los Angeles and Sepulveda VAMCs). Inclusion criteria for the Black Box study were a self-reported and chart-confirmed diagnosis of diabetes, receiving primary care at one of the listed VAMCs, their PCP serving as their primary diabetes care provider, having two or more prior visits with their PCP, and self-reported primary race/ethnicity as Black/African American or White/Caucasian. Inclusion was limited to these race/ethnicities because the objective of the Black Box study was to examine racial differences in patient-provider interactions, and these were the predominant race/ethnicities in the Veterans Affairs population. Exclusion criteria were having dementia or severe mental illness.

Participating patients and PCPs provided written, informed consent. Patients received a $20 honorarium. This study was approved by the Department of Veterans Affairs Central Institutional Review Board.

We audio-recorded one routine primary care visit per patient between October 2017 and January 2020. Visits were selected for audio-recording by consecutively sampling upcoming PCP visits for patients who met the Black Box study inclusion criteria described above. A digital audio-recording device was placed in an unobtrusive site within the exam room, and activated, prior to the recorded visit. Recordings were transcribed verbatim.

There were 421 Black Box study participants. For this analysis, we additionally excluded participants who were not using a hypoglycaemia-causing medication (n = 146 excluded), who did not complete an audio-recorded PCP visit (n = 31 excluded), and whose PCP visit was for an urgent issue (n = 2 excluded). This yielded an analytic population for this study of 242 patients using hypoglycaemia-causing medications who were seen by 49 PCPs. Diabetes medications were ascertained from the Veterans Affairs (VA) Corporate Data Warehouse, which captures patients' electronic health records and pharmacy data. Patients’ diabetes medications at the time of their audio-recorded visit were defined as those medications with active prescription orders in the 180 days preceding the visit, a strategy previously validated in VA records.^8^ As a small minority of patients received diabetes medications outside of the VA system, we also included patients for whom it was clear from their visit dialogue that they were taking hypoglycaemia-causing medications that were not captured in VA pharmacy data (n = 7).

### Qualitative coding

Two investigators (SJP and KAM) independently coded transcripts for discussions related to hypoglycaemia using a directed content analysis approach.^9^^,^^10^ We began with the coding framework developed in our prior study of hypoglycaemia communication.^7^ During coding, we modified our framework by refining initial codes for clarity and to reflect emerging concepts found in the data. The coders met weekly to compare and discuss their codes, and all differences were reconciled until there was complete agreement.

In the final coding framework (Supplementary Table S1), discussions of hypoglycaemia history included when the clinician asked the patient about hypoglycaemia, or when the patient reported hypoglycemic event(s) unprompted. We coded whether patients reported, or did not report, one or more hypoglycemic event(s) in response to PCPs' questions. Review of patients’ home glucose values without mentioning hypoglycaemia, or when the patient reported concerns about normal glucose values being too low, were coded separately. Details of hypoglycemic events included their context (triggers, timing), frequency, severity (the lowest value or need for assistance or treatment), or hypoglycaemia unawareness. Hypoglycaemia anticipatory guidance included general counselling (hypoglycaemia definition, causes, or sequelae), behaviour change (diet, activity, glucose monitoring, medication administration), treating hypoglycemic events, or avoiding hypoglycaemia when driving. We categorized the language that PCPs used to ask about hypoglycaemia, and the language used by patients to report hypoglycemic events, into groups that emerged from the data.

### Hypoglycaemia prevention outcomes

We examined two outcomes related to hypoglycaemia prevention on a per-visit basis: 1) whether the PCP provided any hypoglycaemia anticipatory guidance, ascertained through qualitative coding as described above, and 2) whether the PCP de-intensified (decreased or stopped) or adjusted the timing of a hypoglycaemia-causing medication. To ascertain medication changes, the two coders independently identified discussions of diabetes medication change in the visit dialogue that were categorized by medication class and the specific change that occurred (decreased, stopped, increased, adjusted medication timing, started new medication).

### Patient and PCP characteristics

PCPs completed a baseline questionnaire on enrollment, and patients completed a questionnaire after the recorded visit. PCPs reported their sociodemographic, professional, and practice characteristics. Patients reported their sociodemographic characteristics and diabetes history. Other patient characteristics were ascertained from the VA Corporate Data Warehouse. Patients' HbA1c level was the most recent value within one year prior to their visit, analyzed in clinically relevant categories: <7.0%, 7.0–7.9%, 8.0–8.9%, ≥9.0%. As a sensitivity analysis, we restricted the analysis to patients with HbA1c tested within 90 days of their visit, which may be more relevant to clinical decisions. Patients’ history of cardiovascular and chronic kidney disease was identified by International Classification of Diseases (ICD) codes in the year prior to the visit using standard algorithms.^11^, ^12^, ^13^ The Charlson Comorbidity Index was applied using ICD and procedure codes as previously adapted for VA data.^14^^,^^15^

### Statistical analysis

The frequency of discussions of hypoglycaemia history and PCP actions for hypoglycaemia prevention were described as the proportion of total visits. The association between the patient's hypoglycaemia history discussed in the visit (hypoglycemic event(s) occurred vs. no events vs. hypoglycaemia not discussed) and each hypoglycaemia prevention outcome (as described in “Hypoglycaemia Prevention Outcomes” above) was analyzed using logistic regression with variance accounting for clustering of patients by PCP. The association between patients' HbA1c level and each PCP action for hypoglycaemia prevention was analyzed using logistic regression with variance accounting for clustering, overall and stratified by the patient's hypoglycaemia history. Each of these analyses was adjusted for insulin use as a potential confounder given that insulin use is associated with hypoglycaemia risk and may affect treatment decisions.^1^ Analyses were conducted using Stata version 14 (StataCorp LP, College Station, Texas, USA).

### Role of the funding source

The funders had no role in the study design, data collection, data analysis, interpretation, writing of the report or decision to submit.

## Results

### Patient and PCP characteristics

Among 242 included patients, the mean age was 66.9 years (SD 9.5 years), 10% were female, 64% used insulin, 40% used a sulfonylurea, and 3% used a meglitinide (Table 1). Patients’ mean HbA1c was 8.2% (SD 1.9%) and 23% had an HbA1c < 7.0%. Most patients had a moderate or severe comorbidity burden by the Charlson index. For 90% of patients, their PCP managed most of their diabetes care. PCPs were primarily physicians (MD or DO), and the majority had been practising for more than 10 years (Supplementary Table S2). We analyzed 242 routine primary care visits (one visit per patient), and diabetes management was discussed in 234 visits (97%); visits solely for urgent issues were excluded. Individual PCPs had a median of 4 audio-recorded visits (range 1–13 visits).

**Table 1:** Characteristics of included patients.

Characteristic	Finding (N = 242)
Age, mean (SD), years	66.9 (9.5)
Age ≥65 years	151 (63.7)
Female sex	24 (10.3)
Race/ethnicity	
Black/African American	133 (56.1)
White/Caucasian	84 (35.4)
Hispanic/Latino	18 (7.6)
Other	2 (0.8)
Diabetes duration	
<5 years	29 (12.4)
6–10 years	57 (24.4)
11–20 years	70 (29.9)
>20 years	78 (33.3)
Metformin use	143 (59.1)
Sulfonylurea use	97 (40.1)
Meglitinide use	6 (2.5)
Insulin use, any type	155 (64.1)
Rapid acting insulin use	83 (34.3)
Thiazolidinedione use	6 (2.5)
DPP-4 inhibitor use	33 (13.6)
GLP-1 receptor agonist use	22 (9.1)
SGLT2 inhibitor use	6 (2.5)
Number of diabetes medications, mean (SD)	2.3 (0.9)
Hemoglobin A1c, mean (SD), %^a^	8.2 (1.9)
Hemoglobin A1c category^a^	
<7.0%	56 (23.1)
7.0–7.9%	70 (28.9)
8.0–8.9%	49 (20.3)
≥9.0%	58 (24.0)
Missing	9 (3.7)
Days between HbA1c test and visit, mean (SD)	84.6 (81.5)
Less than 90 days between HbA1c test and visit	134 (57.5)
Cardiovascular disease^b^	105 (43.4)
Chronic kidney disease^b^	100 (41.3)
Charlson comorbidity index^b^	
1–2 (low comorbidity)	43 (17.8)
3–4 (moderate comorbidity)	90 (37.2)
≥4 (severe comorbidity)	109 (45.0)
Yearly family income	
<$20,000	65 (26.9)
$20,000–$60,000	85 (35.1)
$60,000–$100,000	34 (14.1)
≥$100,000	10 (4.1)
Missing	48 (19.8)
Highest level of education	
Did not complete high school	10 (4.1)
High school diploma or GED	83 (34.3)
Associate degree or 2-year college	88 (36.4)
Bachelors or 4-year college	30 (12.4)
Graduate or professional degree	23 (9.5)
Missing	8 (3.3)
PCP manages most of diabetes care	207 (89.6)

a Most recent HbA1c prior to the visit date within 12 months.

b Based on diagnosis codes within 12 months prior to the visit date.

### Frequency and content of hypoglycaemia history discussions

**Fig. 1:**
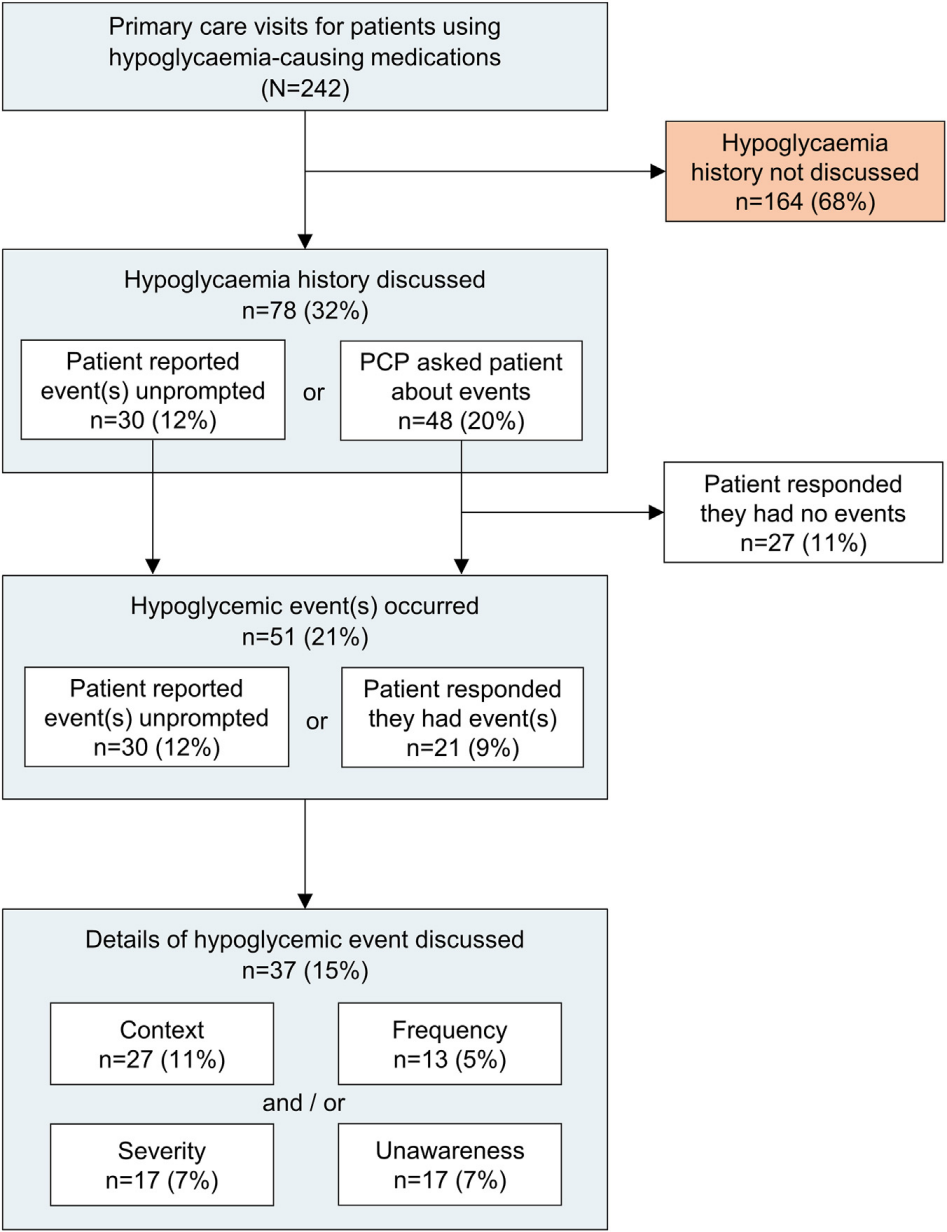
**Frequency and content of hypoglycaemia discussions in primary care visits for patients using hypoglycaemia-causing medications.** All percentages are the subset of total visits for patients using hypoglycaemia-causing medications (N = 242). Details of hypoglycaemic events were discussed in 37 of 51 (73%) visits in which a hypoglycaemic event occurred, which included event context (27/51 visits, 53%), frequency (13/51 visits, 25%), severity (17/51 visits, 33%), and/or hypoglycaemia unawareness (17/51 visits, 33%).

The patient's hypoglycaemia history was discussed in 78 visits (32%, Fig. 1). Hypoglycaemia discussions were more often initiated by PCPs asking about hypoglycaemia (48 visits) than by the patient reporting hypoglycaemia unprompted (30 visits). Patients reported having one or more hypoglycemic event(s) (either in response to PCPs' questions or unprompted) in 51 visits (21%). Within these 51 visits, the context of the hypoglycemic event was discussed in 27 of 51 (53%), or 11% of visits overall. Hypoglycaemia severity was discussed in 17 of 51 visits (33%) with hypoglycemic event(s), or 7% of visits overall. As shown in Supplementary Table S3, 65% of these 17 discussions of hypoglycaemia severity were level 1 hypoglycaemia (glucose 54–69 mg/dl), 24% were level 2 hypoglycaemia (glucose <54 mg/dl), and 6% were level 3 hypoglycaemia (altered mentation or requiring assistance). Hypoglycemic event frequency was discussed in 13 of 51 visits (25%) with hypoglycemic event(s), or 5% of visits overall. Nocturnal hypoglycaemia was discussed in 7 of 51 visits (14%) with hypoglycemic event(s), or 3% of visits overall. Hypoglycaemia unawareness was discussed in 17 of 51 visits (33%) with hypoglycemic event(s), or 7% of visits overall.

Among visits where hypoglycaemia history was not discussed (n = 164), the PCP and patient discussed the patient's home glucose levels in 47 visits. This could have occurred by the patient's self-report, glucometer, or home glucose log, but predominantly by the patient recalling a general glucose range, e.g. *Patient: I'm running 90's, 110. I'm pretty good there,* or a few recent glucose values, e.g. *Patient: This morning was 122. Last night, I think, was 143.* Together, discussions of hypoglycaemia history or home glucose values occurred in 125 total visits (52%).

Patients brought up concerns that glucose values in the normal range were too low in 14 visits (6%). The reasons for these concerns were varied, including the patient being unsure about what blood glucose levels were in the hypoglycemic range, having symptoms they interpreted as hypoglycaemia at normal glucose levels, or where their blood glucose was dropping, and they felt they should intervene.

To examine the range of PCP practices, we analyzed the frequency of hypoglycaemia discussions as proportions of an individual PCP's visits (Supplementary Table S4). Hypoglycaemia history was discussed in mean (SD) of 40% (34%) of individual PCP's clinical visits in the study, and 21 PCPs (43%) discussed hypoglycaemia history in half or more of their visits.

### Language used to assess and report hypoglycaemia

Most of the time (57%) that PCPs asked patients about hypoglycemic events they used the words “low blood sugar” or “lows” (Table 2). In contrast, patients used this language only 26% of the time and were more likely than PCPs to refer to hypoglycaemia using only specific glucose values or hypoglycemic symptoms. Of 31 patient mentions of hypoglycaemia, 26% referred to specific glucose values, e.g. *Patient: I had another 50 the other day, and I got a 45 yesterday*, and 23% referred to only hypoglycemic symptoms, e.g. *Patient: I was starting to feel dizzy. I know my signs*. Within this dialogue, we noted several examples of patients expressing confusion after the PCP asked them about hypoglycaemia, e.g. *PCP: We don't want you to get hypoglycemic. Patient: What's that?* There were also examples of patients denying a history of hypoglycaemia when asked by the PCP while also reporting glucose values < 70 mg/dl in the same visit.

**Table 2:** Language used by primary care providers to assess hypoglycaemia history, and by patients to report hypoglycemic events.

Language category	PCP mentions (N = 65)	Patient mentions (N = 31)	Representative quotes
Low	37 (56.9)	8 (25.8)	PCP: Have you been having low blood sugar? PCP: Any problems with them going too low? Patient: The thing that worries me most is when I hit the low ones. Patient: I've been noticing an increase of like low blood sugar.
Symptoms	5 (7.7)	7 (22.6)	PCP: Do you feel any episodes where you get shaky? Patient: I was starting to feel dizzy. I know my signs. Patient: I'm sitting on my porch the other day and I felt bad and just dizzy like I had drank something.
Low and symptoms	7 (10.8)	1 (3.2)	PCP: Any issues with low sugars? Where you've been sweaty, shaky, had to eat something? Patient: I was at work, casing mail, and I got dizzy. I started sweating. My sugar dropped low.
Specific values	1 (1.5)	8 (25.8)	PCP: Do you ever get into the 60s or the 70s? Patient: I didn't take no insulin, because it was 68. Patient: I had another 50 the other day, and I got a 45 yesterday.
Low and specific values	8 (12.3)	0	PCP: Ever getting the real low ones, down to like 50, 60, like that? PCP: Have you had anything too low? Like you're getting into the 60's 70's range?
Hypoglycaemia or hypo	2 (3.1)	2 (6.5)	PCP: Have you had any episodes of hypoglycaemia? Patient: One day this week, I had a hypo reaction.
Dropping	4 (6.2)	5 (16.1)	PCP: Have you had any episodes where your sugar drops? PCP: Do you drop too much? Patient: They were dropping too much, yeah. Patient: Within the last month, I think it has dropped about maybe five times.
Bottoming out	1 (1.5)	0	PCP: Are you bottoming out at all?

Data are total number of mentions (%), which may occur more than once per visit.

### PCP actions for hypoglycaemia prevention

**Fig. 2:**
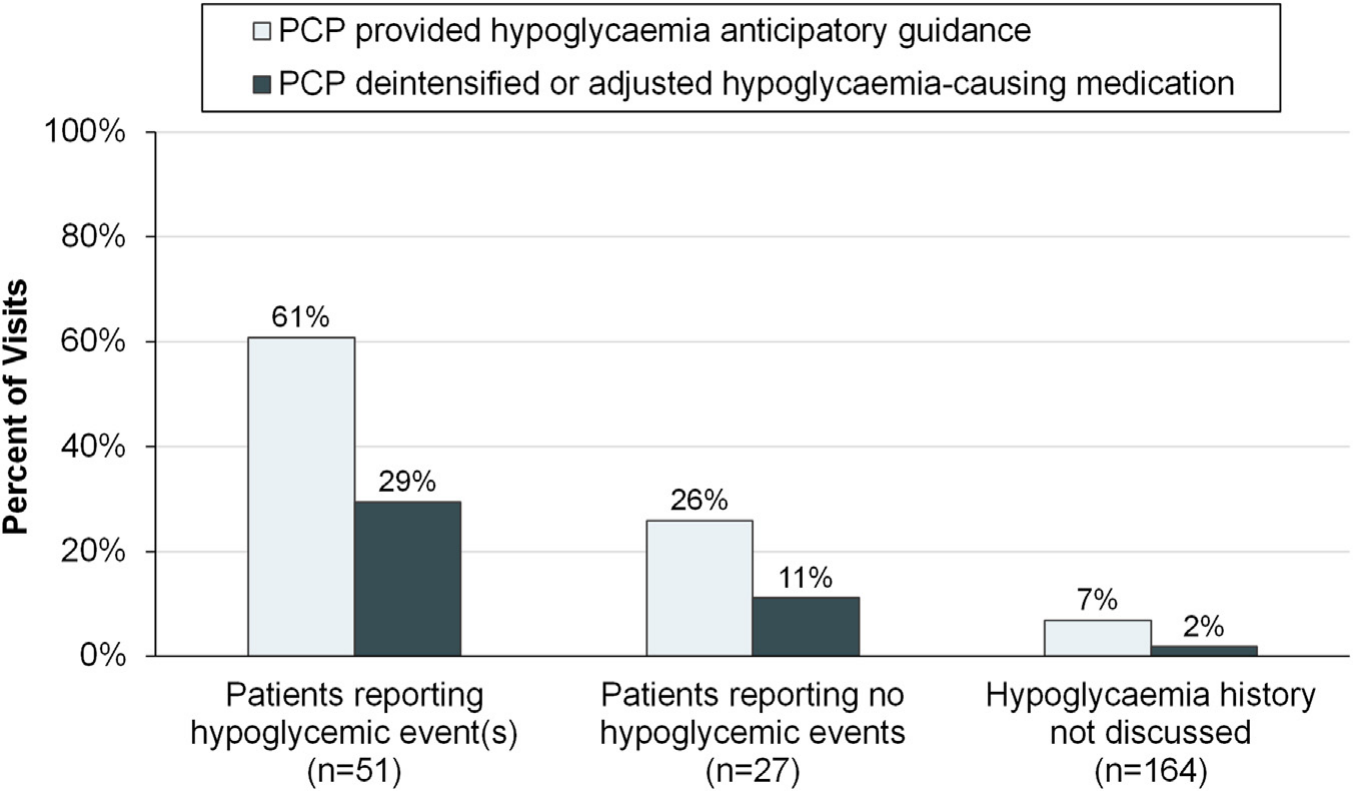
Primary care providers' actions to prevent hypoglycaemia for patients using hypoglycaemia-causing medications, stratified by the patients' hypoglycaemia history.

As shown in Fig. 2, PCPs acted to prevent hypoglycaemia substantially more often when the patient reported hypoglycaemia during the visits, compared to when the patient reported no hypoglycemic events. PCPs rarely acted to prevent hypoglycaemia when hypoglycaemia history was not discussed.

PCPs provided hypoglycaemia anticipatory guidance in 50 visits overall (21%), and in 31 of 51 visits (61%) in which the patient reported hypoglycemic event(s) (p < 0.001 for association between hypoglycaemia history and providing anticipatory guidance). The anticipatory guidance provided included general counselling in 21 visits (9%), behaviour change to prevent hypoglycaemia in 29 visits (12%) and treatment of hypoglycemic events in 13 visits (5%) (Supplementary Table S5). General counselling focused on conveying the danger of hypoglycaemia. Behaviour change counselling focused on holding hypoglycaemia-causing medications while fasting and matching insulin dose to carbohydrate intake; there was no counselling about avoiding hypoglycaemia while driving. Hypoglycaemia treatment counselling was predominantly about carrying glucose tabs; there was no counselling about what steps to take after the initial treatment of a hypoglycemic event, or about glucagon use.

PCPs de-intensified or adjusted the timing of hypoglycaemia-causing medications in 21 visits (9%) overall, and in 15 of 51 visits (29%) in which the patient reported hypoglycemic event(s) (p < 0.001 for association between hypoglycaemia history and medication change). Among the 38 insulin users who had hypoglycemic event(s) (Supplementary Table S6), the most common medication action was to make no change (23 visits, 61%) followed by decreasing the insulin dose (9 visits, 24%). Among the 13 sulfonylurea or meglitinide users who had hypoglycemic event(s), the most common action was to make no medication change (9 visits, 69%) followed by stopping the medication (2 visits, 15%).

### Association between HbA1c and PCP actions for hypoglycaemia prevention

There was no significant association between the patient's HbA1c level and whether the PCP provided hypoglycaemia anticipatory guidance, either overall or stratified by the patient's hypoglycaemia history (Table 3). There was also no significant association between HbA1c and whether the PCP de-intensified or adjusted hypoglycaemia-causing medications. Notably, among 12 patients who reported hypoglycemic event(s) and had HbA1c < 7.0%, hypoglycaemia-causing medications were de-intensified or adjusted in only 5 (42%). Hypoglycemic events reported by patients occurred at a similar frequency across HbA1c levels, although the highest frequency (24% of patients) was among those with HbA1c ≥ 9.0% (Supplementary Table S7). There was no association between PCP assessment of hypoglycaemia and HbA1c level (Supplementary Table S7). Restricting these analyses to the 134 participants with HbA1c tested within 90 days of the visit yielded similar findings to the primary analysis (Supplementary Tables S8 and S9).

**Table 3:** Association between haemoglobin A1c and primary care providers' actions for hypoglycaemia prevention, stratified by the patient's hypoglycaemia history.

	Haemoglobin A1c Category^a^					
	<7.0%	7.0–7.9%	8.0–8.9%	≥9.0%	Missing	p-value^b^
**All patients, N = 242**						
N	56	70	49	58	9	
PCP provided hypoglycaemia anticipatory guidance	11 (19.6)	13 (18.6)	11 (22.5)	13 (22.4)	2 (22.2)	0.99
PCP de-intensified or adjusted a hypoglycaemia-causing medication	7 (12.5)	8 (11.4)	4 (8.2)	2 (3.5)	0	0.30
**Patients reporting hypoglycemic event(s), n = 51**						
N	12	13	9	14	3	
PCP provided hypoglycaemia anticipatory guidance	7 (58.3)	8 (61.5)	7 (77.8)	8 (57.1)	1 (33.3)	0.77
PCP de-intensified or adjusted a hypoglycaemia-causing medication	5 (41.7)	5 (38.5)	3 (33.3)	2 (14.3)	0	0.48
**Patients reporting no hypoglycemic events, n = 27**						
N	5	11	6	5	0	
PCP provided hypoglycaemia anticipatory guidance	2 (40.0)	2 (18.2)	2 (33.3)	1 (20.0)	0	0.78
PCP de-intensified or adjusted a hypoglycaemia-causing medication	1 (20.0)	1 (9.1)	1 (16.7)	0	0	0.81
**Hypoglycaemia history not discussed, n = 164**						
N	39	46	34	39	6	
PCP provided hypoglycaemia anticipatory guidance	2 (5.1)	3 (6.5)	2 (5.9)	4 (10.3)	1 (16.7)	0.83
PCP de-intensified or adjusted a hypoglycaemia-causing medication	1 (2.6)	2 (4.4)	0	0	0	0.69

Data are n (% of column) unless otherwise indicated.

a Most recent value within the past 12 months prior to clinic visit.

b Association between the five HbA1c categories as a nominal variable and the listed PCP action by logistic regression adjusted for insulin use with variance accounting clustering by PCP.

## Discussion

This study identified substantial gaps between guideline-recommended hypoglycaemia prevention practices for patients taking hypoglycaemia-causing medications and what is currently occurring in primary care in multiple centres in the US. While guidelines suggest that hypoglycaemia history should be assessed in each visit, it was only discussed in 32% of visits in this study. PCPs provided anticipatory guidance or made medication changes to prevent hypoglycaemia more often when patients reported a history of hypoglycemic events, although infrequently overall. PCPs de-intensified or adjusted hypoglycaemia-causing medications in fewer than half of patients for whom de-intensification was strongly indicated due to having HbA1c < 7.0% and a history of hypoglycaemia.^2^

The scarcity of hypoglycaemia assessment in primary care is consistent across several lines of evidence. Our prior study that examined audio-recorded primary care visits at one community-based academic practice in 2013–2014 found that hypoglycaemia history was discussed in 24% of visits for at-risk patients.^7^ A single-institution study in 2015 found that hypoglycaemia history was documented in 38% of primary care visit notes for at-risk patients; this study did not evaluate visit dialogue.^16^ In a recent online survey, 39% of patients with insulin-treated type 2 diabetes reported discussing severe hypoglycaemia at every clinic visit.^17^ If we include general discussions of home glucose values as assessment of hypoglycaemia history, there would be a slightly higher rate of assessment in this study. However, discussions of home glucose values were often brief and based on patient recall, and therefore unlikely to detect hypoglycemic events that occurred.

In this study, PCPs rarely acted to prevent hypoglycaemia when hypoglycaemia history was not discussed, suggesting that hypoglycaemia assessment is a necessary step to promote preventive action. A possible solution is to implement standardized assessment of hypoglycaemia history outside of the PCP visit, such as with a hypoglycaemia questionnaire in the patient waiting area or at check-in.^18^ However, the effectiveness of this strategy has not been tested and practice change would be needed to integrate this into the primary care workflow. Continuous glucose monitoring may be another useful tool as it can detect asymptomatic hypoglycemic events, although its use in primary care has been hampered by limited insurance coverage and other barriers.^2^^,^^19^ There is a need for further research to determine the most practical and effective strategies to promote hypoglycaemia assessment in the primary care setting.

We found that the language used by PCPs to ask patients about hypoglycaemia was not understood by some patients, consistent with our prior study.^7^ It is important to determine the language that will most reliably elicit hypoglycaemia when present. Some PCPs in this study asked about hypoglycaemia by giving examples of symptoms or blood glucose values, which may be helpful given that patients predominantly discussed hypoglycemic events in these terms. We also found examples of patients who were concerned about normal blood glucose levels being too low, or who denied hypoglycaemia with blood glucose <70 mg/dl. Together, these findings identify a gap in patient knowledge about the definition of hypoglycaemia that may impede hypoglycaemia assessment and diabetes self-management. The use of patient-centred language by PCPs around hypoglycaemia assessment, prevention, and treatment may help address this problem.

The optimal frequency and detail of anticipatory guidance around hypoglycaemia prevention and treatment have not been defined and should be tailored to individual patients’ history and experience. The hypoglycaemia anticipatory guidance that PCPs provided in this study was generally accurate, although missing key concepts and infrequent. No PCPs counselled patients about the dangers of hypoglycaemia while driving, or to check their blood glucose before driving, which are critical as hypoglycaemia is a preventable cause of fatal motor vehicle accidents.^20^ Counseling about treatment of hypoglycemic events was especially infrequent (only 5% of visits) and narrowly focused on carrying glucose tabs. We found no examples of explicit stepwise instructions for hypoglycaemia treatment such as the “15/15 rule” of ingesting 15 g of carbohydrates and rechecking blood glucose in 15 min.^21^ There was also no counselling about glucagon, despite recommendations in diabetes guidelines for glucagon prescription and caregiver education for all patients at risk for hypoglycaemia. This finding adds to the body of evidence that glucagon is rarely prescribed for adults with diabetes, despite guidelines recommending its use.^22^

Given time constraints in primary care, the opportunity to provide comprehensive hypoglycaemia anticipatory guidance between visits should be explored.^23^ This can be achieved through diabetes self-management education and support (DSMES) programs, which provide accredited diabetes education services and have been shown to improve hypoglycaemia outcomes.^24^^,^^25^ Guidelines recommend DSMES referral for all patients at diabetes diagnosis and with the occurrence of complications including hypoglycaemia.^24^ However, DSMES is utilized infrequently and is not available in many rural and socially disadvantaged areas.^24^^,^^26^^,^^27^ Therefore, expanding DSMES utilization and access may be critical to support hypoglycaemia prevention in primary care.

Diabetes guidelines recommend de-intensifying hypoglycaemia-causing medications when it can be achieved within the patient's individualized glycemic target, especially if there is a history of hypoglycaemia.^2^ We found that de-intensifying or adjusting the timing of hypoglycaemia-causing medications occurred in only 13% of visits for patients with HbA1c < 7.0%, and 42% of those with HbA1c < 7.0% and a history of hypoglycaemia. We focused on the group with HbA1c < 7.0% because that is the recommended glycemic target for many adults with diabetes, although most patients in this study were older adults with at least a moderate comorbidity burden and thus would likely have higher guideline-recommended HbA1c targets.^2^ Prior studies have shown that de-intensifying diabetes medications occurs infrequently, even at lower HbA1c levels and after severe hypoglycemic events.^28^^,^^29^ Our findings highlight the substantial clinical inertia around de-intensifying diabetes medications, even in the context of adverse events reported by the patient to their PCP during the visit. More research is needed to understand how to overcome barriers to de-intensifying diabetes medications when clinically indicated.

Strengths of this study include its multicenter and geographically diverse population, large sample size, and rigorous evaluation of hypoglycaemia assessment and prevention practices through qualitative analyses of visit dialogue. Notably, while there was a large sample size overall, there were relatively few patients in some HbA1c categories, which may have limited statistical power for analyses of associations with HbA1c. We were unable to evaluate diabetes education that patients may have received between clinic visits, although prior research suggests this is infrequent.^24^^,^^27^ As this study used VA data, findings may not be generalizable to all primary care practices, although rates of hypoglycaemia discussions were similar to our prior study in a community-based primary care practice.^7^ It is important to note that VA guidelines and initiatives have focused on reducing rates of hypoglycaemia, and thus may have impacted hypoglycaemia prevention practices.^30^^,^^31^ Nevertheless, hypoglycaemia prevention practices were suboptimal and were similar to our prior study in a different practice setting.^7^

Overall, hypoglycaemia assessment and prevention practices occur infrequently in primary care, and PCPs need additional resources to achieve the level of care recommended in diabetes guidelines. This study supports the need to implement routine hypoglycaemia assessment for at-risk patients, optimize the use of evidence-based diabetes self-management education and support services, and address barriers to de-intensifying hypoglycaemia-causing medications when their risks exceed their benefits. These changes to primary care practice are necessary to improve the safety of diabetes treatment for patients at risk for hypoglycaemia.

## Contributors

Concept and design: SJP, NMM, MCB, SS. Acquisition, analysis, or interpretation of data: SJP, KAM, MCB, JAL, HSG, JTB, DLW, BGB, AT, SS, NMM. Drafting of the manuscript: SJP. Critical revision of the manuscript for important intellectual content: SJP, KAM, MCB, JAL, HSG, JTB, DLW, BGB, AT, SS, NMM. Statistical analysis: SJP. Obtained funding: SJP, SS. Supervision: NMM, MCB, SS. SJP is the guarantor of this work and, as such, had full access to all the data in the study and takes responsibility for the integrity of the data and the accuracy of the data analysis. NMM, MCB, SS, and SJP had access to and verified the data. NMM and SJP were responsible for the decision to submit the manuscript.

## Data sharing statement

Data for this project include protected health information considered sensitive by the United States Veterans Health Administration. Data can be shared pursuant only to a valid request through the US Freedom of Information Act.

## Declaration of interests

SJP received honoraria from the American Diabetes Association (ADA) for speaking at the ADA 2022 Scientific Sessions, the ADA 2023 Clinical Update Conference; for authoring the ADA Making Technology Work module on hypoglycaemia; and for reviewing the ADA Diabetes Is Primary CE Certificate program.
