# Supplementary material for: Assessment and prevention of hypoglycaemia in primary care among U.S. Veterans: a mixed methods study
Source: Lancet Reg Health Am. 2023 Nov 24;28:100641. doi: 10.1016/j.lana.2023.100641 (PMC10701452; doi:10.1016/j.lana.2023.100641)
Supplement: Supplementary Tables [file mmc1.pdf]

Supplementary Material

Supplementary Table 1. Coding framework for hypoglycemia communication

Supplementary Table 2. Characteristics of primary care providers caring for patients included in this study

Supplementary Table 3. Categorization of the severity of hypoglycemia among the 51 visits in which the patient reported hypoglycemic event(s)

Supplementary Table 4. Frequency and representative quotes of the hypoglycemia anticipatory guidance given by primary care providers

Supplementary Table 5. All changes to diabetes medications in visits for included patients, stratified by insulin use and whether the patient reported hypoglycemia in the visit

Supplementary Table 6. Association between hemoglobin A1c and hypoglycemia history and events

Supplementary Table 7. Association between hemoglobin A1c and primary care providers' actions for hypoglycemia prevention, restricted to patients with HbA1c tested within 90 days of the visit

Supplementary Table 8. Association between hemoglobin A1c and hypoglycemia history and events, restricted to patients with HbA1c tested within 90 days of the visit

Supplementary Table 1. Coding framework for hypoglycemia communication

Code category	Code description	Notes
Hypoglycemia history	Clinician asked about hypoglycemic event(s)	Included are discussions where the clinician asked directly about hypoglycemia, or where this was implied through symptoms or glucose values. Not included are discussions of blood glucose levels without mention of whether the patient was having hypoglycemia, or patient reports of concern about non-hypoglycemic blood glucose levels being too low.
	In cases where the clinician asked about hypoglycemic event(s), the patient responded that they had one or more hypoglycemic event(s)	Included are patient reports that they had blood glucose <70 mg/dl, had an event that they interpreted as hypoglycemia, or had characteristic symptoms that were suspected to be hypoglycemia in the context of the visit. The intent is to include events that occurred in the interval between this visit and the last visit; not included are events that occurred in the distant past. Not included are events where the patient reported feeling hypoglycemia symptoms but measured blood glucose ≥70 mg/dl.
	Patient reported hypoglycemic event(s) unprompted	Included are hypoglycemic events using the same definition as above.
Details of hypoglycemic event(s)	The context around specific hypoglycemic event(s) was discussed	Included are discussions of what triggered the event, when it occurred, or any other contextual details.
	The severity of hypoglycemic event(s) was discussed	Severity is defined as the lowest blood glucose value in the context of hypoglycemia, or the need to use corrective action or obtain the help from another person. Not coded are reports of what the blood glucose value was during a single hypoglycemic event (without indicating whether that was the lowest value).
	The frequency of hypoglycemic event(s) was discussed	Frequency is defined as how often hypoglycemic events are occurring. Discussions limited to the timing of single hypoglycemic event(s) are not included.
	Nocturnal hypoglycemia was discussed	Includes discussion of whether hypoglycemia occurs at night, vivid dreams, nightmares, morning headaches, or sudden awakenings. Not coded for a patient reporting a single episode of hypoglycemia that occurred at night. Note, this category was analyzed as a subcategory of hypoglycemic event context.
	Awareness of hypoglycemia was discussed	Includes assessment of the blood glucose level at which the patient feels hypoglycemic symptoms, or whether they have symptoms at low blood glucose levels. Not included are discussions where the patient reported a symptomatic hypoglycemic episode without discussion of whether they do or do not have symptoms typically.
Hypoglycemia anticipatory guidance	General counseling	Includes discussion of the definition of hypoglycemia, its causes and sequelae, or its relationship to diabetes treatment.
	Behavior change to prevent hypoglycemia	Includes discussion of changes in diet, activity, blood glucose monitoring, medication administration, or other behaviors intended to prevent hypoglycemic events from occurring.
	Treatment of hypoglycemic events	Includes ingesting carbohydrates, eating a meal, carrying glucose tabs, rechecking blood glucose after treatment, using glucagon, or following-up with a clinician after hypoglycemic events.
	Avoiding driving with hypoglycemia	Includes discussions where the clinician counseled the patient on avoiding driving with hypoglycemia, or when they are at risk of hypoglycemia, or when blood sugar may be falling.
Other topics	Home glucose values reviewed	Includes discussions of the patient's home glucose values by their self-report, or on a diary or their glucometer. More than a single value must be included, not just the highest value. Not included are discussions of the patient's hemoglobin A1c level.
	Patient concerned that normal glucose values are too low	Includes discussions were the patient reported concerns about non-hypoglycemic blood glucose values (≥70 mg/dL) being too low.
	Diabetes medication change	Includes any changes to diabetes medications that were discussed during the visit.

Supplementary Table 2. Characteristics of primary care providers caring for patients included in this study

Characteristic	Finding (N=47)*
Age, mean (SD), years	46·8 (9·1)
Female gender	30 (65·2)
Race / ethnicity	
Black / African American	9 (19·2)
White / Caucasian	19 (40·4)
Hispanic / Latino	1 (2·1)
Asian	13 (27·7)
Other	1 (2·1)
No response	4 (8·5)
Degree	
MD or DO	41 (87·2)
NP	5 (10·6)
PA	1 (2·1)
Years in practice	
<6 years	8 (17·0)
6-10 years	7 (14·9)
11-20 years	18 (38·3)
>20 years	12 (25·5)
No response	2 (4·3)
Hours per week in direct patient care	
6-10 hours	4 (8·5)
11-20 hours	3 (6·4)
21-40 hours	25 (53·2)
>40 hours	14 (29·8)
No response	1 (2·1)

* Patients in this study were cared for by 49 primary care providers; data are available for the 47 who completed the provider survey

Supplementary Table 3. Categorization of the severity of hypoglycemia among the 17 visits in which hypoglycemia severity was discussed

**Hypoglycemia severity**	**Visits (N=17)**
Level 1 (glucose 54-69 mg/dl)	11 (64·7)
Level 2 (glucose <54 mg/dl)	4 (23·5)
Level 3 (altered mental state or requiring assistance)	1 (5·9)
Severity unable to be determined from dialogue	1 (5·9)

Supplementary Table 4. Frequency of hypoglycemia discussions and preventive actions, analyzed as the proportion of an individual primary care provider's (PCP's) visits in which they occurred

	Proportion of an individual PCP's visits in which the event occurred			Event occurred in ≥50% of visits for the individual PCP, N (%)
**Event**	**Mean (SD)**	**Median (IQR)**	**Range, min – max**	
Hypoglycemia history was discussed	0·40 (0·34)	0·33 (0·60)	0 – 1	21 (42·9)
PCP asked patient about events	0·26 (0·32)	0·14 (0·33)	0 – 1	10 (20·4)
PCP provided hypoglycemia anticipatory guidance				
Overall (N=49 PCPs)	0·24 (0·28)	0·14 (0·33)	0 – 1	11 (22·5)
For patients reporting hypoglycemic event(s) (N=13 PCPs)	0·46 (·42)	0·67 (0·67)	0 – 1	7 (53·9)
PCP deintensified or adjusted hypoglycemia-causing medication				
Overall (N=49 PCPs)	0·13 (0·23)	0 (0·2)	0 – 1	4 (8·2)
For patients reporting hypoglycemic event(s) (N=13 PCPs)	0·53 (0·44)	0·5 (1·0)	0 – 1	7 (53·9)

Supplementary Table 5. Frequency and representative quotes of the hypoglycemia anticipatory guidance given by primary care providers

Type of anticipatory guidance	Visits	Representative quotes
General counseling	21	PCP: [Hypoglycemia] is when your blood sugar gets really low and you get dizzy and then you need to eat food. Yeah so I don't want that to happen to you.<br><br>PCP: It is a little safer that you have one high than one low. I mean, low we are talking 60 is dangerous. Okay?<br><br>PCP: If we overdo it, if I blast you with 100 units of insulin any kind at one time, like if I were to shoot you with that right now, I'd probably drop your blood sugar significantly. That's what insulin does. It brings these numbers down.<br><br>PCP: What kills people with diabetes in an abrupt way is if someone has a dangerously low blood sugar all of a sudden. They could have a seizure, something like that.
Behavior change to prevent hypoglycemia	29	PCP: So if you're eating something, you should be taking insulin, but I certainly think it's reasonable for you to reduce the dose of insulin with less calorie-dense foods.<br><br>PCP: Well, sometimes an evening snack will help to forestall that nighttime drop in your sugar.<br><br>PCP: Okay, so, that medicine that you take for the sugar, the glipizide, you cannot take that medicine and not eat because it will drop your sugar.
Treatment of hypoglycemic events	13	PCP: You have those chewable tablets, right? Make sure you have that on hand.<br><br>PCP: I want you to carry around those [glucose] tablets and I hope you never have to use them.

Supplementary Table 6. All changes to diabetes medications in visits for included patients, stratified by insulin use and whether the patient reported hypoglycemia in the visit

		Hypoglycemia history			
**Diabetes medication change**	**Overall**	**Patient reported hypoglycemic event(s)**	**Patient reported no hypoglycemic events**	**Hypoglycemia history not discussed**	**p-value***
**Insulin users**	**n=155**	**n=38**	**n=17**	**n=100**	
No change	120 (77·4)	23 (60·5)	11 (64·7)	86 (86·0)	0·002
Increased insulin dose	14 (9·0)	2 (5·3)	3 (17·7)	9 (9·0)	0·40
Decreased insulin dose	11 (7·1)	9 (23·7)	1 (5·9)	1 (1·0)	0·004
Adjusted insulin instructions	3 (1·9)	1 (2·6)	1 (5·9)	1 (1·0)	0·48
Increased sulfonylurea dose	1 (0·7)	0	0	1 (1·0)	†
Stopped sulfonylurea	2 (1·3)	2 (5·3)	0	0	†
Increased other medication dose	1 (0·7)	0	1 (5·9)	0	†
Decreased other medication dose	1 (0·7)	1 (2·6)	0	0	†
Stopped other medications	2 (1·3)	0	1 (5·9)	1 (1·0)	†
Started new medication	3 (1·9)	1 (2·6)	0	2 (2·0)	0·81
**Sulfonylurea users**	**n=87**	**n=13**	**n=10**	**n=64**	
No change	77 (88·5)	9 (69·2)	8 (80·0)	60 (93·8)	0·036
Increased sulfonylurea dose	2 (2·3)	0	1 (10·0)	1 (1·6)	†
Decreased sulfonylurea dose	3 (3·5)	1 (7·7)	1 (10·0)	1 (1·6)	0·37
Stopped sulfonylurea	2 (2·3)	2 (15·4)	0	0	†
Started new medication	3 (3·5)	1 (7·7)	0	2 (3·1)	0·45

Columns may not sum to 100% because more than one change could happen in a given visit. No meglitinides were changed. New medications started were long-acting insulin (n=2), metformin (n=1), DPP-4 inhibitor (n=2), and thiazolinedione (n=1).

* Association between hypoglycemia history categories and medication change by logistic regression accounting clustering by PCP

† Too few outcomes to calculate p-value

Supplementary Table 7. Association between hemoglobin A1c and hypoglycemia history and events

	Hemoglobin A1c Category*					
	**<7·0%**	**7·0-7·9%**	**8·0-8·9%**	**≥9·0%**	**Missing**	**p-value†**
N	56	70	49	58	9	
Hypoglycemia history discussed	17 (30·4)	24 (34·3)	15 (30·6)	19 (32·8)	3 (33·3)	0·99
PCP asked patient about hypoglycemia	12 (21·4)	15 (21·4)	11 (22·5)	8 (13·8)	2 (22·2)	0·74
Patient reported event(s) unprompted	5 (8·9)	9 (12·9)	4 (8·2)	11 (19·0)	1 (11·1)	0·21
Hypoglycemic event(s) were reported (in response to a PCP question or unprompted)	12 (21·4)	13 (18·6)	9 (18·4)	14 (24·1)	3 (33·3)	0·75

Data are n (% of column) unless otherwise indicated

* Most recent value within the past 12 months prior to clinic visit

† Association between the five HbA1c categories as a nominal variable and the listed component of hypoglycemia history by logistic regression adjusted for insulin use with variance accounting clustering by PCP

Supplementary Table 8. Association between hemoglobin A1c and primary care providers' actions for hypoglycemia prevention, restricted to patients with HbA1c tested within 90 days of the visit

	Hemoglobin A1c Category*				
	**<7·0%**	**7·0-7·9%**	**8·0-8·9%**	**≥9·0%**	**p-value†**
All patients, n=134					
N	34	37	30	33	
PCP provided hypoglycemia anticipatory guidance	4 (11·8)	8 (21·6)	6 (20·0)	6 (18·2)	0·82
PCP deintensified or adjusted a hypoglycemia-causing medication	4 (11·8)	6 (16·2)	3 (10·0)	1 (3·0)	0·35
Patients reporting hypoglycemic event(s), n=24					
N	5	7	4	8	
PCP provided hypoglycemia anticipatory guidance	2 (40·0)	5 (71·4)	3 (75·0)	3 (37·5)	0·35
PCP deintensified or adjusted a hypoglycemia-causing medication	2 (40·0)	4 (57·1)	2 (50·0)	1 (12·5)	0·39
Patients reporting no hypoglycemic events, n=15					
N	2	7	4	2	
PCP provided hypoglycemia anticipatory guidance	1 (50·0)	2 (28·6)	1 (25·0)	1 (50·0)	0·47
PCP deintensified or adjusted a hypoglycemia-causing medication	1 (50·0)	1 (14·3)	1 (25·0)	0	0·09
Hypoglycemia history not discussed, n=95					
N	27	23	22	23	
PCP provided hypoglycemia anticipatory guidance	1 (3·7)	1 (4·4)	2 (9·1)	2 (8·7)	0·82
PCP deintensified or adjusted a hypoglycemia-causing medication	1 (3·7)	1 (4·4)	0	0	0·88

Data are n (% of column) unless otherwise indicated

* Most recent value within the past 90 days prior to clinic visit

† Association between the four HbA1c categories as a nominal variable and the listed PCP action by logistic regression adjusted for insulin use with variance accounting clustering by PCP

Supplementary Table 9. Association between hemoglobin A1c and hypoglycemia history and events, restricted to patients with HbA1c tested within 90 days of the visit

	Hemoglobin A1c Category*				
	**<7·0%**	**7·0-7·9%**	**8·0-8·9%**	**≥9·0%**	**p-value†**
N	34	37	30	33	
Hypoglycemia history discussed	7 (20·6)	14 (37·8)	8 (26·7)	10 (30·3)	0·52
PCP asked patient about hypoglycemia	6 (17·7)	10 (27·0)	7 (23·3)	4 (12·1)	0·28
Patient reported event(s) unprompted	1 (2·9)	4 (10·8)	1 (3·3)	6 (18·2)	0·05
Hypoglycemic event(s) were reported (in response to a PCP question or unprompted)	5 (14·7)	7 (18·9)	4 (13·3)	8 (24·2)	0·84

Data are n (% of column) unless otherwise indicated

* Most recent value within the past 90 days prior to clinic visit

† Association between the four HbA1c categories as a nominal variable and the listed component of hypoglycemia history by logistic regression adjusted for insulin use with variance accounting clustering by PCP
